# Immunopathogenic and Neurological Mechanisms of Canine Distemper Virus

**DOI:** 10.1155/2012/163860

**Published:** 2012-11-04

**Authors:** Otávio Valério Carvalho, Clarisse Vieira Botelho, Caroline Gracielle Torres Ferreira, Paulo Oldemar Scherer, Jamária Adriana Pinheiro Soares-Martins, Márcia Rogéria Almeida, Abelardo Silva Júnior

**Affiliations:** ^1^Instituto de Veterinária (IV), Universidade Federal Rural do Rio de Janeiro, Rodovia BR 465, km 7, 23890-000 Seropédica, RJ, Brazil; ^2^Laboratório de Virologia Molecular Animal (LVMA), Departamento de Veterinária, Universidade Federal de Viçosa, Avenida Peter Henry Rolfs, s/n, Campus Universitário, 36570-000 Viçosa, MG, Brazil; ^3^Department of Microbiology and Molecular Genetics, Medical College of Wisconsin, Milwaukee, WI 53226, USA

## Abstract

Canine distemper is a highly contagious viral disease caused by the canine distemper virus (CDV), which is a member of the *Morbillivirus* genus, Paramyxoviridae family. Animals that most commonly suffer from this disease belong to the Canidae family; however, the spectrum of natural hosts for CDV also includes several other families of the order Carnivora. The infectious disease presents worldwide distribution and maintains a high incidence and high levels of lethality, despite the availability of effective vaccines, and no specific treatment. CDV infection in dogs is characterized by the presentation of systemic and/or neurological courses, and viral persistence in some organs, including the central nervous system (CNS) and lymphoid tissues. An elucidation of the pathogenic mechanisms involved in canine distemper disease will lead to a better understanding of the injuries and clinical manifestations caused by CDV. Ultimately, further insight about this disease will enable the improvement of diagnostic methods as well as therapeutic studies.

## 1. Introduction

Canine distemper is an infectious disease caused by a member of the *Morbillivirus* genus, Paramyxoviridae family, infecting a broad range of terrestric and aquatic carnivores. Canine distemper virus (CDV) is an enveloped virion which contains a nonsegmented single-stranded negative-sense RNA genome that encodes six structural (nucleocapsid N, matrix M, fusion F, hemagglutinin H, phospho-P and large-L proteins) and two nonstructural (C and V proteins) proteins [1].

CDV has been reported in dogs, ferrets, wild dogs, foxes, jackals, coyotes, hyenas [2], lions, tigers, leopards, cheetahs [3], seals, sea lions, and dolphins [4]. The domestic dog is the most suffered species, and, although the disease has been also found in big cats, CDV has not been detected in domestic cats. However, experimental infection with SPF cats revealed that this species can sustain CDV replication with pronounced lymphopenia, without displaying any clinical signs [5, 6].

The *Morbillivirus *genus includes other important highly infectious pathogens like measles (MV) and rinderpest viruses (RPV), and almost all members present equivalent tropism and tissue distribution in their respective hosts. Morbilliviruses are transmitted by aerosols and produce clinical similarities, such as fever, serous nasal discharge, and cough, as well as respiratory and gastrointestinal signs often complicated by secondary bacterial infections. Furthermore, the most notorious property of morbillivirus infection is the establishing of severe transitory immunosuppression [7, 8].

RPV and pest des petits ruminants virus (PPRV) are the only morbilliviruses known to naturally induce clinical disease in artiodactyls; however, infected meat feeding may develop subclinical infection in dogs, which is confirmed by the production of immunoglobulins to RPV [9]. Resembling the susceptibility of cattle and buffalo herds upon virulentRPV infection, CDV infection in ferrets is more severe than in dogs leading to their death within two to three weeks [10]. Irregular morbillivirus outbreaks in marine mammals exhibit a critical course of disease [4]. On the other hand, MV infections in humans and nonhuman primates, CDV infections in dogs, and PPRV infections in goats and sheep present lower degrees of severity [11–13].

Ferrets infected with CDV develop all of the disease signs described in MV human syndrome, including fever, rash, immunosuppression, and gastrointestinal and respiratory involvement; however, CDV infection usually results in the death of these animals. Clinical and molecular similarities among these viruses makes the CDV-infected ferret an ideal model for understanding the CDV and consequently MV pathogenesis and immunosuppression [7, 14].

Canine distemper clinical signs include anorexia, depression, conjunctivitis, hyperkeratosis of digital cushions, catarrhal inflammation of bronchi and larynx, vomiting and diarrhea, and intense pustules in the abdomen and thighs [15]. When the nervous system is affected, apathy, ataxia, paraplegia, quadriplegia, muscular atrophy, myoclonus, vocalization, tremor, incontinence, night time seizures, coma, dryness of the retina, circling, constant crying, and blindness are observed [6]. 

Canine distemper disease presents a variable progression which can lead the animal to either develop a restricted or a full set of clinical signs, depending on the virus strain that caused the disease [16]. Immunization with live attenuated or recombinant canary poxvirus expressing H and F proteins vaccines has been used to protect dogs; however, outbreaks of canine distemper are still reported worldwide. In the absence of CDV-specific and efficient antiviral drugs, only supportive treatment is performed for infected animals [6, 17]. Given the seriousness of injuries produced by CDV, it is critical to gain more insight about the factors involved in the pathogenesis of immunological and neurological paths in order to search for therapeutic strategies that will solve or prevent the disease. Thus, studies that focus on immunopathogenesis and neurological mechanisms of CDV are extremely important. 

## 2. Infection and Viral Distribution 

Under natural conditions of exposure, CDV is usually transmitted by aerosols and infects the upper respiratory tract. During the first 24 hours post-infection, viral replication occurs in macrophages and circulating B and T cells, and later on, viral particles spread through the lymphatic pathway to bronchial lymph nodes and tonsils [18, 19]. Primary viral replication in the lymphoid tissues leads to a lasting and severe immunosuppression [20, 21]. 

About two to four days after infection there is an increased amount of viral particles in tonsils as well as retropharyngeal and bronchial lymph nodes; however, there is a small number of infected mononuclear cells in other lymphoid organs. During the first four to six days post-infection, viral replication occurs in the lymphoid system, bone marrow, thymus, spleen, lymph nodes, mesenteric lymph nodes, Peyer's patches, stomach cells, Küpffer cells, and mononuclear cells around the bronchial and pulmonary vessels. Then, between the second and sixth days, hyperthermia is observed due to the high rate of viral multiplication at the lymphoid organs as well as leukopenia caused by the depletion of lymphoid cells [22]. 

After viremia, about eight to ten days post-infection, CDV disseminates via hematogenous pathways or cerebrospinal fluid (CSF) to several epithelial tissues and central nervous system (CNS) [19]. Infected lymphocytes infiltrated in epithelia may locally release massive virus amounts favoring epithelial cell entry and consequently, respiratory, intestinal, and urinary infection [14]. According to de Swart et al. [23], T lymphocyte and dendritic cell (DC) infiltration in the dermis of MV-infected macaques is responsible for the dermatological manifestations.

Studies have used recombinant CDV expressing green fluorescent protein (eGFP-CDV) in experimental infections of ferrets to identify the host cells supporting morbillivirus infection. At first, eGFP-CDV replied strongly in circulating B and T cells, then infected lymphoid cells in lymph nodes, spleen, mucosa-associated lymphoid tissue (MALT) and thymus, and finally spread to epithelial cells throughout the body [14]. While at initial infection mainly lymphatic tissues and organs were targeted, the virus later spread to glial cells and neurons anterogradely via the olfactory nerve and hematogenously through the choroid plexus and cerebral blood vessels [24].

The signaling lymphocyte activation molecule (SLAM) or CD150, which is expressed on activated T cells, B cells, monocytes, and DCs [25, 26], is a lymphotropic receptor for morbilliviruses [27, 28]. Attachment of morbilliviruses to susceptible cells occurs through the interaction of SLAM and the viral H protein, one of the two glycoproteins that are inserted into the viral membrane and soon after it will be expressed on the surface of infected cells. Upon virus-ligand interaction, H protein undergoes structural changes and transmits a signal to the F protein, which serves as a mediator to assist the viral and host cell membranes fuse with one another. However, the fusogenicity, growth characteristics, and cell tropism depend predominantly on the viral H protein [29, 30].

It has been demonstrated that SLAM is a very efficient cellular receptor for wild type CDV strains in established Vero cells stably expressing canine SLAM. Also, immunocytochemistry studies have shown a very limited SLAM expression in the CNS when compared to the lymphoid tissues. Therefore, it is likely that there are other viral receptors [19, 31]. 

Another receptor known as Nectin 4/PVLR4 was recently identified as the epithelial cell receptor for MV. This protein is a single pass type I transmembrane protein, a member of the poliovirus receptor-like proteins (PVRLs), which are adhesion receptors of the immunoglobulin superfamily [32, 33]. Nectins can modulate many cellular functions, including cell movement, and have been described as entry receptors for several other viruses [34, 35]. 

PVRL4 is expressed at moderate levels in normal airway epithelial cells but is highly upregulated on the surfaces of adenocarcinoma cells of the lung, breast, and ovary. Because PVRL4 is a cellular marker found in several classes of human cancers, MV could potentially be used to specifically infect cancer cells and promote oncolytic effects by turning the immune system against tumors [36].

Most viruses use receptors to enter hosts or spread through their epithelial barriers; however, recent studies suggest that MV targets nectin-4 to emerge in the airways and release into the environment late in infection instead of subsidizing the early stages of infection [36, 37]. Whereas CDV is a close relative of MV and CDV uses CD150/SLAM as a receptor to infect lymphocytes and DCs, this virus possibly also uses Nectin 4/PVRL4 to infect epithelial cells, since these cells do not express SLAM [38].

CDV can be found in cells of the respiratory, urinary, and gastrointestinal tracts, endocrine cells, lymphoid tissues, nerve cells, vascular fibroblasts and keratinocytes, CNS, different subgroups of lymphoid cells, as well as in bronchial, endothelial, and neuroectodermal cells [39, 40].

The most common illness manifestations are pyrexia, anorexia, nasal discharge, conjunctivitis, diarrhea, and skin pustules with hyperkeratosis. Neurological signs appear either during the system phase of the disease, after, or in the lack of it, and those signs are predominantly responsible for the death of infected animals [18].

## 3. Immunosuppression and Protective Immunity 

In addition to respiratory and gastrointestinal signs, canine distemper is characterized by severe leukopenia and loss of lymphocytes ability to proliferative. This results in immunosuppression and increases host susceptibility to opportunistic infections, which is the main cause of deaths associated with morbillivirus [15, 41].

In fact, characterization of events that lead to suppression of the immune system is a subject of intense study. All morbilliviruses show a high lymphotropism, which correlates with the SLAM protein in subsets of lymphocytes [42, 43]. SLAM binding is needed for lymphocyte infection, viral spread, and immunosuppression in CDV-infected ferrets [43]. 

Apoptosis in cells of the immune system, followed by immunosuppression induced by morbillivirus is considered one of the main reasons for severe leukopenia [44, 45]. After viral elimination from peripheral blood, decrease in antigen presentation and lymphocyte maturation may contribute to the continuation of the immunosuppressant status while lymphoid organs get repopulated [15]. The MV N protein has been correlated with measles-induced immunosuppression by modulating antigen presentation at DCs and subsequent interfering on T cells function, and the same immunosuppressive strategy may be applicable in CDV infection [46, 47].

During the acute phase of canine distemper, lymphopenia is characterized by transient depletion of CD4+ T cells, CD8+ T cells, and CD21+ B cells from peripheral blood [15]. CD4+ T cells are the most often target cells during the acute CDV infection, and the CD4+ reduction in lymphoid organs exceeds the CD8+ T cells depletion [48, 49]. CD4+ lymphocytes are rapidly depleted for several weeks, probably due to virus-induced apoptosis [50, 51], whereas CD8+ cells are less affected and recover relatively fast [52].

A reduced number of circulating cells of the immune system might be the result of deficient cell production in the primary and secondary lymphatic organs, and also due to apoptosis of peripheral blood leukocytes. The observation of programmed cell death in lymphoid tissues can occur in a substantial number of uninfected cells, indicating the existence of an additional apoptosis mechanism independent of viral action [50, 53]. Other mechanisms besides apoptosis directly induced by viruses, such as hyperactivity of the innate immune system or apoptosis of lymphoid cells through the Fas protein, must be considered as possible mechanisms of cell death in canine distemper [53, 54]. 

The host defense depends on the innate immune system, which is also responsible for producing signals that activate the adaptive immune response [55]. Type I interferons (IFN I, e.g., IFN *α*/*β*) are critical elements in the innate immune defense against viruses [56, 57]. However, *in vitro* study with a highly virulent CDV strain showed interference with IFN function by disrupting signaling for the synthesis of antiviral proteins [58].

At the initial course of CDV infection there is an increase of viral proteins, messengers RNAs (mRNAs), major histocompatibility complex class II (MHC II), hyaluronate CD44 receptors, and proinflammatory cytokines [59, 60]. Chronic injuries are characterized by reduction or loss of viral proteins and mRNAs, a strong expression of MHC II and massive influx of monocytes [49]. 

The resolution of CDV infection requires critical humoral and cellular immune responses. The magnitude of the humoral immune response is related to the intensity of the disease. In general, humoral protection in canine distemper occurs by producing antibodies against viral nucleoproteins, followed by the development of specific humoral immune response to viral envelope proteins [61, 62]. Absence of an effective humoral response leads to an acute clinical status, which is usually fatal. The measurable specific antibodies against the virus produced at 10 to 14 days post-infection lead to either viral persistence or elimination. The latter depends directly on the specificity of immunoglobulin against viral envelope proteins [63].

Cellular immune response is critical in MV clearance; however, humoral immune response is relevant for subsequent MV RNA clearance and limitation of remitting viral infection [64]. On the other hand, CDV clearance succeeds later in the inflammatory phase via neutralizing antibodies and complement-mediated humoral cytotoxity, critical factors for the suppression of viral particles, and for clinical recovery in infected animals [65, 66].

Cellular immune response in recovering CDV-infected dogs can only be identified during a short time interval, however, a vigorous and continue cellular immunity involving cytotoxic T, killer, and natural killer (NK) cells can determine the CDV elimination in infected animals [11, 67]. Effector cellular immunity against CDV is prominent in the lack of a measurable humoral immune response [15].

Specific antibodies for H protein prevent development of injuries in the CNS of infected animals [62]. However, the temporary absence of antibodies against viral M protein and attenuated or delayed response of complement-fixing antibodies against viral envelope proteins result in persistence of the neurological disease [61, 63].

Neutralizing antibodies and humoral cytotoxicity mediated by the complement represent critical factors to eliminate free viral particles as well as for clinical prognosis of infected animals [11–65]. Neutralizing antibodies are responsible for preventing intra and extracellular viral dissemination; however, prolonged exposure to antibodies results in internalization of viral antigens from cellular membranes. Then, due to reduction of viral spread, humoral modulation to viral antigens expression can represent a favorable factor for persistent infection in dogs, probably because of both decrease in recognition of antigens and inadequate cytotoxicity of mediated by the complement [68, 69]. 

Specific humoral immune response to CDV can be detected over dogs' lives while the cellular immune response can only be detected for a short time in convalescent dogs [11]. The importance of cellular immune response in canine distemper is evidenced through protective immunity in the absence of humoral response mediated by measurable antibodies [70].

CDV may persist in regions of the white matter or peripherally to those, since they are absent of demyelinating inflammatory lesions [18]. Viral persistence occurs during progressive development of the chronic disease; in this case, viral replication happens more rapidly compared to the immune response. Pathogenesis of lesions in the chronic phase is directly related to viral activity [19, 71, 72].

In dogs over six years, viral persistence in the CNS after acute infection may trigger old dog encephalitis, a rare and chronic form of panencephalitis, which is characterized by progressive inflammation of the grey matter in both brain hemispheres and brainstem. This condition involves immunocompetent animals with viral persistence in the CNS and immune response marked by increased expression of MHC. Injuries are present in the cerebral cortex, basal nucleus, thalamus, and hypothalamus and they are also characterized by disseminated lymphocytic perivascular infiltration, diffuse proliferation of microglial cells pervasive, astrogliosis, neuronal degeneration, and neuronophagia [73].

## 4. Mechanisms of Development of Neurological Lesions 

### 4.1. CDV Entry and Spread in CNS

Infection of the CNS is the most serious complication of canine distemper, leading to a variety of neurological disorders often with a bad prognosis. Usually, neurologic signs occur in the absence of systemic signs [19, 74]. 

Aspects such as the presence of antibodies and deposition of immune complexes, which occur during systemic immune response, can contribute to viral spread in the CNS endothelium. Both free and platelet- or lymphocyte-bound viruses invade vascular endothelial cells in the meninges, choroid plexus cells of the fourth ventricle, and ependymal cells in the linear ventricular system [11]. Moreover, frequent subpial and periventricular lesions associated with the fact that the virus is easily found in the cells of the choroid plexus and ependyma demonstrate that viral penetration in the brain tissues occurs through the CSF [18, 75].

Astrocytes are considered target cells for CDV in the CNS and they are related to demyelinating lesions which are very characteristic of acute and chronic encephalitis [71, 76]. Regarding viral dispersal in the CNS, it was observed that spread of CDV in astrocytes did not require infectious particles, since the viral H protein was crucial to enable viral transmission cell to cell through intercellular fusion activity. Furthermore, it was found that viral spread to neighboring cells may occur in very short intervals of time, introducing the idea about lateral transmission between cells in the brain be highly efficient. Therefore, CDV might use gap junctions to spread to the astrocytes syncytial network [77].

While the acute phase of canine distemper is completely devoid of effective antiviral immune response, IgM antibodies anti-CDV arise within the first two weeks of infection [78]. Despite the absence of perivascular cuffing, many CD8+ cells are found in the acute demyelinating lesions and also diffusely distributed in the brain parenchyma, which is correlated with areas of viral infection. Elevated titers of IL-8 are found in the cerebrospinal fluid of animals with acute injuries in the myelin. This suggests that initial activation of microglia can elicit invasion of T cells in the CNS [79].

Direct action of viral activity in the CNS causes acute encephalitis, which appears in the early stages of the infection in young or immunosuppressed animals [73, 80]. CDV causes multifocal lesions in the gray and white matter. Lesions in the gray matter include neuronal infection and necrosis and may result in polioencephalomalacia. On the other hand, damages in the white matter are defined by myelinic lesions and are correlated with viral replication in glial cells. Whether there is a physiological immaturity of the immune system and/or immunosuppression induced by the virus inflammatory changes are minor [18, 80].

In ferrets, CDV can also use neuronal pathways to access and disseminate through the CNS [24]. However, infection of astrocytes in the white matter happens exclusively in dogs and it is very important to the process of demyelination. Such cells are the main target of CDV *in vivo *and *in vitro* [77]. Newly discovered morbilliviruses, phocine distemper virus (PDV), porpoise morbillivirus (PMV), and dolphin morbillivirus (DMV) have also been shown to produce multifocal demyelination mechanism in infected animals [81].

In most cases of spontaneous canine distemper the virus causes multifocal lesions in the gray and white matter in the CNS [73]. Usually demyelinating lesions prevail and lesions in the gray matter are absent. Preferential sites are the cerebellum and periventricular white matters, especially around the fourth ventricle, optical pathways and spinal cord [75]. 

CDV reaches the brain through infected mononuclear cells that cross the blood-brain barrier. Another more important possibility to be considered is the fact that CDV can also reach the CNS via circulation in the cerebrospinal fluid and associates with the ependyma that coats the ventricles [75]. This explains the frequent periventricular and subpial location of the lesions [19]. 

### 4.2. Neuropathology of Demyelinating Processes

CDV has tropism for myelinated tissues of the brain and spinal cord in the CNS. In contrast to other neurological infections, neurons are not primarily affected in canine distemper disease. Therefore, both the distribution and nature of injuries diverge from those observed for most other encephalitis cases [82].

Demyelination begins about three weeks after infection and it is enabled by severe immunosuppression and an absence of inflammatory process [19], since perivascular cuffing is not observed [83]. Initial lesions are characterized by the swelling of myelin layers and astrocytes, vacuolization of the white matter, as well as phagocytosis of myelin. An apparent explanation for demyelination would be oligodendrocyte infection; however, under optical microscopy it has been observed that the majority of infected cells in the white matter are astrocytes [84].

Most electron microscopy studies agree that infection of oligodendrocytes is very rare in canine distemper [75, 85, 86]. In spite of that, mRNA from CDV can be detected in approximately 8% of the oligodendrocytes around lesions. In canine brain cell cultures an early infection with viral transcription, but not translation, was observed strictly in oligodendrocytes, resulting in a noncytolytic infection and slow propagation of a virulent CDV strain [87].

**Figure 1:**
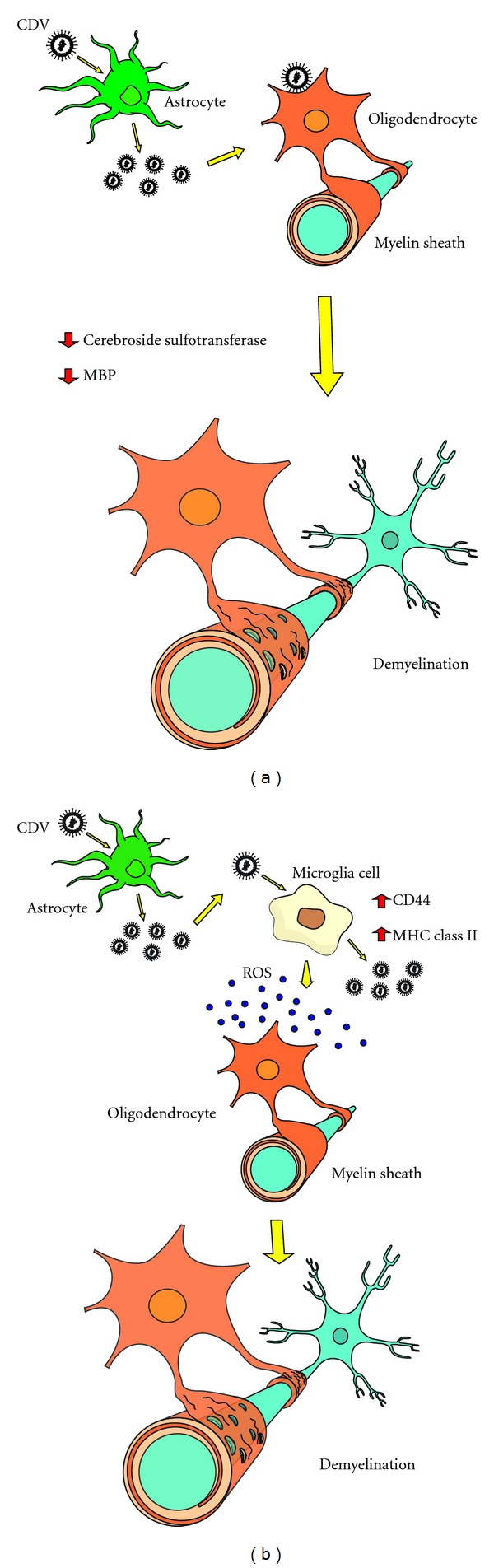
Schematic representation of the possible mechanisms involved in the demyelination process in the course of acute canine distemper disease. (a) Primary demyelination: direct action of CDV on oligodendrocytes. (b) Secondary demyelination: indirect action of CDV on oligodendrocytes and myelin sheath. CDV: canine distemper virus; PBM: myelin basic protein; ROS: reactive oxygen species (free radicals).

There are two hypotheses about the mechanisms involved in the process of demyelination during the acute phase of infection by CDV. The first hypothesis describes the direct damage of the myelin or myelinogenic cells (primary demyelination) (Figure 1(a)). Studies of ultrastructure showed microvacuolation and loss of organelles in infected oligodendrocytes. These morphological transformations are preceded by metabolic dysfunction with a pronounced decrease of cerebroside sulfotransferase activity (specific enzyme of oligodendrocytes) soon after infection [88]. Also, a dramatic decrease in myelin transcription in infected brain cell cultures has been demonstrated. Overall, acute infection of white matter results in metabolic changes leading to demyelination of oligodendrocytes [19]. 

The second hypothesis outlines myelinic degeneration as a following outcome during viral infection (secondary demyelination) (Figure 1(b)). It has been shown in demyelination studies [89] that CDV was able to stimulate microglia cells resulting in diffuse MHC II and adhesion molecule CD44 expression upregulation in the white matter, which may be related to demyelination pathogenesis in the acute phase of canine distemper disease. Ultimately, activation of microglia cells produces release of toxic factors such as reactive oxygen species (ROS) and proteolytic enzymes, and increased phagocytic activity that may possibly contribute to oligodendrocyte damage and/or destruction of the myelin sheath [19, 89, 90].

With regard to demyelination induced by viral disease in animal models, inflammatory lesions in the white matter are derived at least partly from the intrathecal immune response against CDV, which predominantly infects astrocytes [19, 72]. However, previous studies examined inflammatory lesions in the white matter of infected dogs and it has been shown that CDV possesses the ability to spread to other areas of the central nervous system (CNS) causing new injuries [19]. Therefore, viral persistence is the driving force behind the progression of the disease [91].

Parameters such as virus strain as well as age and immune competence of the infected animal are critical to determine how considerably affected the CNS is. Necrosis in the nervous tissue occurs with greater frequency in young and immunodeficient animals infected by CDV. Demyelinating lesions are more prevalent in adult dogs and immunocompetent animals [92]. Encephalitis caused by CDV can be described in four ways: (1) encephalitis in young dogs, as acute and severe, with multisystemic manifestations, including neurological ones; (2) encephalitis in adult dogs, as chronic, with possibility of casual neurological signs; (3) old dog encephalitis; (4) chronic, relapsing encephalitis, the latter with minor occurrence [93].

Demyelinating leukoencephalitis represents the primary consequence of canine distemper in dogs. As a result of morphology in the course of neuropathological transformations, this disease has been considered an important animal model for spontaneous occurrence of human demyelinating disease, which is analogous to multiple sclerosis (MS) [15, 77]; the most important human demyelinating inflammatory disease. Epidemiological data suggests an infectious cause for MS however it remains uncertain [94].

Young dogs are commonly affected by acute encephalitis during CDV infection and systemic clinical signs usually are present. Histopathological examination of animals up to six months old and positively diagnosed with CDV through RT-PCR showed both acute and severe multifocal demyelinating encephalitis and necrotic lesions in the white matter [80]. Usually, histopathological findings in canine distemper are described as demyelinating lesions in the white matter, necrosis foci, and presence of inclusion bodies in astrocytes and perivascular cuffing [15].

Multifocal encephalitis involves chronic infectious features in adult dogs aged between four to six years, without precedence or association to systemic clinical signs of canine distemper. Lesions caused by continuous demyelination and disseminated mononuclear infiltration are induced by MHC expression, which is noticeable in all microglial cells, even though viral antigen is restricted to a small number of astrocytes. Transformations occur through both hyperplasia of astrocytes and microglial proliferation in subpial and subependymal structures in the white matter. Interaction of antibodies to infected macrophages leads to injuries in the CNS by stimulation of macrophage activation, which releases oxygen free radicals and causes destruction of oligodendrocytes and myelin sheath [22, 73, 80].

Dogs testing positive for CDV aged over twelve months may show chronic encephalitis [80]. When infected animals in this age range were examined, multifocal lesions were found with intense demyelination, which is indicative of chronic and severe multifocal demyelinating encephalitis, and it was also aggregated to eosinophilic intranuclear corpuscles in astrocytes. Furthermore, an increase of antibody titers against CDV was detected in animals with such lesions, and usually they did not present systemic clinical signs [92, 95, 96].

Depending on recovery level and rate of the immune system, animals can either become quickly debilitated or recover after developing a mild or subclinical disease. On the other hand, considering an intermediate group, animals can have either a partial or slow recovery and tend to develop chronic or even relapsing illness [97, 98], with progression of demyelinating lesions as a result of immunopathologic reactions [99, 100].

## 5. Conclusion

Canine distemper is an important viral disease that affects dogs and other carnivores in a multisystemic manner. Canine distemper virus (CDV) infects many cell types including epithelial, mesenchymal, hematopoietic, and neuroendocrine cells from various organs and tissues. Main clinical manifestations include respiratory and gastrointestinal signs, immunosuppression, and demyelinating leukoencephalitis. The early destruction of lymphocytes mediated by the virus results in immunosuppression of infected animals; however, subsequent mechanisms that affect antigen presentation as well as lymphocyte maturation are still poorly understood. In the central nervous system (CNS), CDV infection causes severe demyelinating lesions which are still not elucidated. Regardless of its cause, demyelination occurs through two main processes: damage of the myelin itself or myelinogenic cells (primary demyelination) or axonal injury which promotes a secondary effect in the myelinic degeneration (secondary demyelination).
